# Theranostic Nanomedicine for Malignant Gliomas

**DOI:** 10.3389/fbioe.2019.00325

**Published:** 2019-11-14

**Authors:** Michele d'Angelo, Vanessa Castelli, Elisabetta Benedetti, Andrea Antonosante, Mariano Catanesi, Reyes Dominguez-Benot, Giuseppina Pitari, Rodolfo Ippoliti, Annamaria Cimini

**Affiliations:** ^1^Department of Life, Health and Environmental Sciences, University of L'Aquila, L'Aquila, Italy; ^2^Department of Biology, Sbarro Institute for Cancer Research and Molecular Medicine, Temple University, Philadelphia, PA, United States

**Keywords:** theranostic nanoplatform, brain tumors, targeted therapy, drug delivery, diagnosis

## Abstract

Brain tumors mainly originate from glial cells and are classified as gliomas. Malignant gliomas represent an incurable disease; indeed, after surgery and chemotherapy, recurrence appears within a few months, and mortality has remained high in the last decades. This is mainly due to the heterogeneity of malignant gliomas, indicating that a single therapy is not effective for all patients. In this regard, the advent of theranostic nanomedicine, a combination of imaging and therapeutic agents, represents a strategic tool for the management of malignant brain tumors, allowing for the detection of therapies that are specific to the single patient and avoiding overdosing the non-responders. Here, recent theranostic nanomedicine approaches for glioma therapy are described.

## Theranostic technology

**Figure 1:**
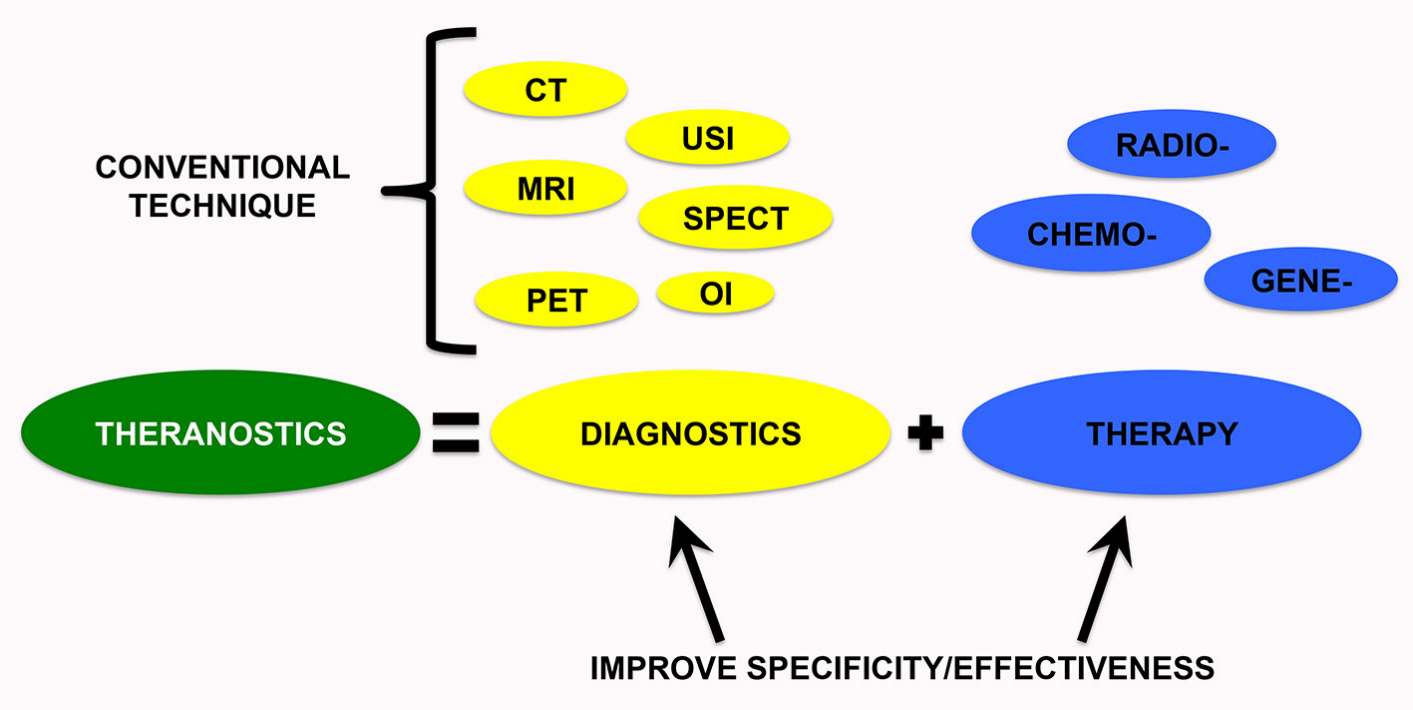
Theranostic medicine provides new tools to improve diagnostic specificity and therapeutic effectiveness. Therefore, a nanoparticle-containing tracer can be useful to overcome the limitations of conventional diagnostic and therapeutic techniques. CT, computed tomography; MRI, magnetic resonance imaging; USI, ultrasound imaging; OI, optical imaging; PET, positron emission tomography; SPECT, single-photon emission computed tomography.

Theranostics is the combination of the two terms “Therapeutics” and “Diagnostics,” referring to technologies that include both diagnostic and therapeutic applications (Figure 1). The interest in personalized medicine, and thus, theranostic approaches used for individualized diagnosis and treatment are gaining increasing attention (Sun, 2010; Kelkar and Reineke, 2011; Kievit and Zhang, 2011; Ahmed et al., 2012; Wang Y. et al., 2014). This technology allows us to save time and decrease costs but, notably, also allows us to contain side effects of a single strategy (Lammers et al., 2010), obtaining better patient outcomes (Duncan, 2003; Peer et al., 2007).

Nanoplatforms (NPS) are nanoparticles combined with drug and molecular imaging probes, including metal nanoparticles, polymer-drug conjugates, polymer micelles, liposomes, and dendrimers. NPS show several advantages over conventional formulations, allowing for the conjugation or entrapment of drugs (Peer et al., 2007). Nanoparticles (NPs) are complex drug delivery systems, which can be structurally divided into the internal layer (core), and external layer (shell). Nanodimensions ensure that nanoparticles are able to increase drug solubility, mitigate cytotoxicity, and improve drug pharmacokinetic profiles. The creation of nanoplatforms, combining drugs with molecular probes, increases the drug half-life in the circulatory system, and specifically, delivers anticancer drugs to target tissues, controlling the drug release through detectors responsive to different stimuli such as pH, temperature, light, ultrasound, and enzymatic activities, thus improving the delivering of the required drug concentration to the area of interest (Tong and Cheng, 2007). By means of improving the circulating half-life, nanomedicines can accumulate in tumors through the Enhanced Permeability and Retention (EPR) effect (Golombek et al., 2018). In the last years, it has been reported that EPR varies among mouse models and patients, between tumor types of the same origin, and also among tumors and metastases of the same patient (Harrington et al., 2001; Tanaka et al., 2017), thus explaining the heterogeneous outcomes of nanomedicine clinical trials. To overcome this issue, efforts should be focused on the use of methods that can be employed to individualize and improve nanomedicine treatments.

Despite the advantages offered by the EPR effect, the passive targeting approach offers a limited benefit in the treatment of gliomas and other CNS disorders. In these situations, the BBB remains impenetrable for different nanostructures that tend to accumulate in off-target tissues that also have vasculature gaps, such as the liver, or lymph nodes (Nam et al., 2018).

Since theranostic approaches require the use of molecular imaging tools, a combination of drug delivery systems with imaging techniques, such as computed tomography (CT), magnetic resonance imaging (MRI), optical and ultrasound (US) imaging, positron emission tomography (PET), and single photon emission computed tomography (SPECT), are currently under study. All these imaging techniques, using sensible and specific probes, can, in fact, assess drug efficacy during the drug development procedures (Cai et al., 2006; Pysz et al., 2010; Ai, 2011; Ang et al., 2014), optimizing the right choice of imaging tools and agents (Jokerst and Gambhir, 2011) and defining the best combination for specific therapeutic applications.

## Multifunctionalized Nanoplatforms

Multifunctionalized NPS are promising therapeutic approaches in cancer therapy (Table 1). Indeed, they offer numerous advantages over conventional agents, including specific targets, the higher ability to solubilize hydrophobic or labile drugs, lower systemic toxicity (resulting in better pharmacokinetics and higher potential to image), and better treatment and prediction of a therapeutic response. NPS utilize nanostructures, such as nanoparticles, made from soluble or colloidal aqueous solutions and with sizes ranging between 10 and 100 nm (Bhojani et al., 2010). The small size allows them to pass via blood capillaries and reach the specific tumor cells (Bhojani et al., 2010). They have the advantages of modifying the nature and the number of linkers on and within the surface of a nanoparticle and its dimensions, thus leading to the control of the loading/releasing of the entrapped or covalently linked drugs. The NPS can also ameliorate the efficacy of current drugs or tracers, triggering a selective delivery. Among NPS, those based on nanovesicles are also biocompatible, thus increasing the maximum tolerated dose of the drug with low toxicity. This leads to an increased concentration of the agent inside the tumor and a simultaneous decrease in side effects (Liong et al., 2008; Bhojani et al., 2010). The entrapment of the drug with nanoplatforms reduces the limit for the use of poorly soluble or poorly absorbed agents by encapsulating them in the matrix of the NPS during the design and synthetic processes. Furthermore, the encapsulation prevents premature degradation of drugs or inactivation during plasma transport. Being a multidelivery system represents one of the most advantageous characteristics of NPS. They can carry imaging tracers, targeting ligands, therapeutic agents, and “cloaking” agents that avoid interference with the immune system (reviewed in Bhojani et al., 2010; Mendes et al., 2018).

**Table 1:** Nanoplatforms examples and their characteristics.

	**Nanoplatforms**	**Biosafety**	**Size**	**Loading capacity**	**References**
Magnetic Nanoparticle	DOX IONPS	Low toxicity	10–50 nm	High	Choi et al., 2012
Polymer-conjugates	DOX-GEM GADOLINIUM HPMA	Low toxicity	20–70 nm	Good	Vilos and Velasquez, 2012
Nanovesicles	β(CD) SPIO Polymeric micelles	Really low toxicity	10–70 nm	High	Liong et al., 2008; Bhojani et al., 2010
Dendrimers	asODN MNP PAMAM	Potential toxicity	10–40 nm	High	Pan et al., 2007

*Different nanoplatforms and the respective biosafety, size, and loading capacity*.

*DOX-conjugated, doxorubicin; PAMAM, poly(amidoamine); IONPs, iron oxide nanoparticles; DOX-GEM, gemcitabine (GEM), and doxorubicin (DOX); HPMA, N-2- hydroxypropylmethacrylamide; asODN, antisense oligodeoxynucleotides; MNPs, Magnetic Nanoparticles; SPIO, superparamagnetic iron oxide*.

Cancer researchers have shown high interest in theranostic approaches, particularly to detect and develop a solid nanosystem strategy for cancer treatment and diagnosis that can be translated into clinical practice (Cole et al., 2011).

Typical examples of the design of biocompatible nanoplatforms used as theranostic agents are based on magnetic nanoparticles, polymers, vesicles nanoparticles, and dendrimers.

Magnetic nanoparticles have been prepared using, for example, IONPs (iron oxide nanoparticles) coated with a human serum albumin. This formulation is referred as a biocompatible material for a chemotherapeutic agent, photosensitizers, and NPS (reviewed in Choi et al., 2012). Polymeric conjugates for drug delivery, biodistribution, and drug efficiency were extensively investigated (Vilos and Velasquez, 2012). Lammers et al. (2010) synthesized a simultaneous delivery of doxorubicin and gemcitabine, and they were labeled with a gadolinium HPMA (N-2-hydroxypropylmethacrylamide) copolymer to investigate the biodistribution of nanotheranostics using an MRI (Lammers et al., 2010). This investigation reported that tumor-targeted polymeric drug vectors could be utilized to deliver two different chemotherapeutic drugs to tumors concurrently (Lammers et al., 2010).

Polymeric micelles, nano core/shell structures constituted by amphiphilic copolymers, were thoroughly tested as theranostic carriers and imaging probes as well. The amphiphilic block copolymers captured the superparamagnetic iron oxide (SPIO) or Mn-SPIO nanoparticles and are employed for the MRI (Lu et al., 2009; Liu et al., 2011; Xie et al., 2011; Su et al., 2013). β-cyclodextrin (β-CD) has been successfully used to encapsulate SPIO nanoparticles and small molecule anticancer drugs (Su et al., 2013). Several multifunctional polymeric micelles for tumor drug delivery and distribution have been designed, with particular attention to the creation of a well-controlled nanostructure. The use of polymeric micelles is advantageous because they can entrap an elevated number of hydrophobic drugs and contrast agents, maintaining their hydrophilic feature as a carriers, compared to liposomes or soluble polymers. Polymeric micelles are recognized as multifunctional delivery systems that are able to maximize therapeutic efficacy (Vilos and Velasquez, 2012).

Liposomes are already approved by the FDA since they are able to incorporate drugs, such as chemotherapeutics (Al-Jamal and Kostarelos, 2011). Approved formulations are liposomal doxorubicin and pegylated liposomal doxorubicin, which show low toxicity, cardiac safety, and less alopecia, myelosuppression, nausea, and vomiting when compared to conventional anthracyclines. The difficulty in releasing the encapsulated drug in the target area is caused by a limit in the liposome system. To overcome this issue, new liposome systems have been designed that are able to induce a pH and temperature response or the activation of certain enzymes on liposome cavities, thus improving the drug release in the targeted area (Lindner and Hossann, 2010; Wang D. et al., 2014). Recently, multifunctional theranostic nanoplatforms, using contrast agents encapsulated with liposomes, have been developed for the simultaneous diagnosis of early stage of disease and drug delivery, utilizing liposomes that encapsulate contrast agents, resulting in the creation of multifunctional NPS (Kenny et al., 2011; Na et al., 2011; Petersen et al., 2012). A theranostic nanosystem that provides the incorporation of magnetic nanoparticles inside the liposomes has been developed (Fattahi et al., 2011). Thus, multifunctional theranostic liposomes are widely used in treatment and for the detection of diseases, and they represent a valid carrier to further improve the diagnostic and therapeutic efficacy.

Furthermore, dendrimers are gaining increasing importance in the theranostic field as they can, due to their size, encapsulate several drugs or imaging tracers with high efficiency. For instance, dendrimers can bind non-covalently or covalently to chemotherapeutic drugs, imaging agents, and other biologically active targeting moieties, such as peptides, monoclonal antibodies, and folates (Boas and Heegaard, 2004; Mintzer and Grinstaff, 2011; Lo et al., 2013). The characteristic structure of dendrimers can stabilize the hydrophobic nanoparticles through the ligand exchange reaction method. Recently, multifunctional doxorubicin (DOX)-conjugated poly(amidoamine) (PAMAM) dendrimers have been developed with a specific platform for targeted chemotherapy that uses pH to release the drug to tumor cells. This multifunctional dendrimer presented excellent biocompatibility, biodistribution, and satisfactory cancer imaging results (Chang et al., 2011). Dendrimers represent promising structures for functionalization and also for conjugation with drugs (chemotherapeutics and imaging tracers) and DNA/RNA (Pan et al., 2007; Merkel et al., 2010; Zottel et al., 2019).

## Gliomas

Glioma is a common type of tumor arising from glia-supporting neurons. About 33% of all brain tumors are gliomas and show different malignancy and differentiation grades. Symptoms depend on the area of the brain affected and by the degree of malignancy; they include headaches, nausea, or vomiting, speech difficulties, vertigo, and motor alteration. In its advanced stages, seizures may be a common manifestation. Gliomas are classified on the basis of the glial type but also on the genetic signature that predicts the outcome and the response to treatment. Gliomas are classified, according to the World Health Organization, as astrocytoma, anaplastic astrocytoma and glioblastoma, oligodendrogliomas, ependymomas, and mixed gliomas (Wesseling and Capper, 2018). Glioblastoma (GB) multiforme is the most malignant and common (more than 60%) type of primary astrocytomas (Rock et al., 2012). Despite the modern therapies to treat GB, it is still a deadly disease with an extremely poor prognosis. Patients usually have a median survival of ~14–15 months from the diagnosis (Thakkar et al., 2014). The standard treatment for GB is the resection of the tumor by neurosurgery, followed by radiation, and chemotherapy administration. However, these therapies are often ineffective, having a high rate of recurrence and drug resistance over time, accompanied by severe neurological deterioration of the affected patient (Silantyev et al., 2019).

The surgical approach is often not efficient due to the frequent persistence of tumoral foci; this leads to the recurrence of the disease (Alphandéry et al., 2015) thanks to the high proliferative rate and invasive behavior of GB cells. In this regard, several studies have reported the crucial role of bulk removal in increasing life expectancy and patient outcome (Silantyev et al., 2019). However, even bulk removal is not completely efficient since it is, generally, followed by relapses. For these reasons, GB is considered a not treatable disease. Temozolomide (TMZ) is currently the gold standard treatment for GB. Its metabolites form a complex with alkyl guanine alkyl transferase (O6 MGMT- DNA repair protein), leading to DNA damage; however, some patients show resistance to TMZ. Thus, many studies have reported the efficacy of the combination of TMZ with different compounds, such as curcumin, resveratrol, O6-benzylguanine, valproic acid, anti-epilectic drugs, interferon 1-β, mesenchymal stem cells, and anti-malarial drugs [extensively reviewed in Bahadur et al. (2019)], with reduced resistance and increased treatment efficacy. In particular, it has been reported that the combined administration of bone marrow-derived mesenchymal cells (MSCs), interferon β (IF-β), and TMZ significantly decreased tumor progression *in vitro* and increased the survival of patients following synergistic effects *in vivo* (Park et al., 2015). More recently, the simultaneous administration of the inducer of autophagy, sirolimus, the inhibitor of autophagy, Chloroquine, and TMZ on glioblastoma cells was investigated in order to obtain lysosome disruption and apoptotic death (Hsu et al., 2018). In the same way, several new molecules were proposed to enhance TMZ activity in glioblastoma both *in vitro* and *in vivo* (extensively reviewed in Bahadur et al., 2019).

The diagnostic tools to detect brain tumors are represented by imaging tests, mainly MRI, including different specialized MRI scan components, including functional MRI, perfusion MRI, and magnetic resonance spectroscopy. These tools help us to understand tumor size and to plan treatment. Other imaging exams may include PET, a computerized tomography (CT) scan, and a cerebral angiogram. Molecular testing of the tumor could also be recommended for the identification of specific proteins, genes, and other factors (i.e., tumor markers) distinctive to the tumor. Indeed, some biomarkers may help in determining a patient's prognosis, increasing the chance of recovery. For the final and definite diagnosis, a biopsy of the tumor's tissue is usually necessary in order for it to be analyzed by a pathologist (Piquer et al., 2014; Tandel et al., 2019).

The first occurrence in tumor transformation is not completely clarified. However, it seems that the genetic signature is different in grade II gliomas, astrocytoma, and oligodendroglioma. All tumors initially show the same invasive phenotype, making it difficult to develop a unique therapy. Progression-associated genetic modifications target cell cycle-controlling pathways and growth promoting, causing focal hypoxia, necrosis, and angiogenesis. Retinoblastoma protein (Rb) mutation was identified in 20% of malignant gliomas (Behin et al., 2003), although gliomas may also contain mutations in other molecules involved in Rb signaling, including cyclin-dependent kinase (CDK) and the cell cycle regulator cyclin-dependent kinase inhibitor 2A, multiple tumor suppressor 1 (p16^INK4A^). Most of the anaplastic astrocytoma show homozygous mutation, deletion, and promoter hypermethylation in the INK4A/ARF locus that encodes two tumor suppressors [p16^INK4a^ and an alternate reading frame tumor suppressor, p14^ARF^ (Yamanaka, 2008)]. Moreover, it has been shown that PDGF (platelet-derived growth factor) and platelet-derived growth factor receptor (PDGFR) signaling are involved at the beginning of the progression from astrocytoma to GB. In fact, elevated levels of PDGFRα have been reported in all types of gliomas, particularly in GB. Also, gliomas induce the overexpression of other mitogens, including IGF-1 (Insulin like Growth factor) and EGF (Epidermal growth factor) as well (Wong et al., 1992; Chakravarti et al., 2002; Nicholas et al., 2006; Puputti et al., 2006; Newton, 2010). Their receptors are present as constitutively active mutant forms in gliomas (Wong et al., 1992), leading to the activation of numerous pathways, including PI3K/AKT PBK, phospholipase protein C, and RAS/mitogen-activated protein kinase. In turn, these pathways control invasion, cell proliferation, apoptosis, and differentiation processes (Schlessinger, 2000). A common alteration (20–40%) identified in glioblastoma that affects the PI3K-Akt pathway is the genetic loss or mutation of the tumor suppressor gene PTEN (Phosphatase and Tensin homolog deleted on chromosome ten). Indeed, PTEN is a key negative regulator of the PI3K/Akt pathway (Stambolic et al., 1998; Cantley and Neel, 1999). In addition, gliomas display the upregulation of angiogenic factors, such as the FGF (fibroblast growth factor), TGF (transforming growth factor), Interleukin 8 (IL-8), and Vascular-Endothelial Growth Factor (VEGF) (Benoy et al., 2004; Slettenaar and Wilson, 2006; Xiao et al., 2018). The combination of the genetic alteration of these factors triggers a malignant glioma with an aggressive phenotype and that is resistant to intensive therapies. In this tumorigenic process, glioma stem cells exert a leading role (Uchida et al., 2000; Gaya et al., 2002; Kondo et al., 2004; Gürsel et al., 2011). Since glioma stem cells are able to self-propagate, in order to avoid recurrence, it is fundamental to target specifically them (Kroonen et al., 2008). The new possibility to isolate GBM stem cells allows for new therapeutic approaches, among which are gene replacement, knockdown, or silencing (Kroonen et al., 2008). Since each GB patient shows a peculiar molecular profile, the response at radio- and chemotherapies is different. On this basis, different GB cell lines may show a different response to Cdk inhibitors (Caracciolo et al., 2012; Cimini et al., 2017).

GB, and other solid tumors as well, encounter metabolic reprogramming; thus, the tumor is able to survive in hypoxic conditions and sustain angiogenesis and hyperproliferation (Kroemer and Pouyssegur, 2008; Tennant et al., 2009; Fidoamore et al., 2016; Antonosante et al., 2018).

In particular, tumor cells activate the glycolytic pathway, also in the presence of oxygen (Warburg effect) (Frezza and Gottlieb, 2009). Indeed, tumor cells exploit the glycolytic signaling intermediates for anabolic reactions (Gatenby and Gillies, 2004). Only the cells subjected to these alterations are able to survive in the tumor environment, suggesting the presence of a selection of those with the altered metabolic phenotype (Tennant et al., 2009). The progress in the genetic biology of gliomas, and the recent insertion of manipulable experimental models, allows for the development of effective targeted therapy.

## Targeted Theranostic Nanoplatforms for Brain Cancer Therapy and Imaging

The human brain is an extremely complicated organ, which simultaneously regulates and supervises several functions. Successful therapy in brain cancers is restricted because the administered therapeutic entity cannot reach the targeted area after systemic administration (Cheng et al., 2014), and this is the main obstacle for the transport of the therapeutic agents represented by the blood–brain barrier (BBB). The BBB consists of a physical barrier, composed by vascular endothelial cells, and is held together by tight junctions, transporters, receptors, enzymes, and the ATP-dependent, 170-kDa efflux pump P-glycoprotein (Sonali et al., 2016a,b,c). The BBB retains the passage of agents with a molecular weight >500 Da but also of the majority of small sized molecules (Wei et al., 2014; Agrawal et al., 2017a,b). ATP-binding P-gp at the same time exerts the efflux function for xenobiotics, and their strong expression inhibits the passage of substrates through the BBB. The majority of the chemotherapeutics are hydrophobic and larger in molecular size; thus, they cannot cross the BBB spontaneously. Also, chemotherapeutics are substrates of multidrug-resistant drug efflux pumps, which are active on both tumor vascular cells and the BBB (Zong et al., 2014).

Brain cancers are difficult to detect and treat during the primary stages. The diagnosis and the detection of the volume of the brain cancers are complex because an accumulation of extracellular fluid (Koo et al., 2006) surrounding the tumor region is generally present. Since the 1970s, the primary modality to treat brain cancer includes surgical resection and/or chemotherapy or radiotherapy (Koo et al., 2006).

**Figure 2:**
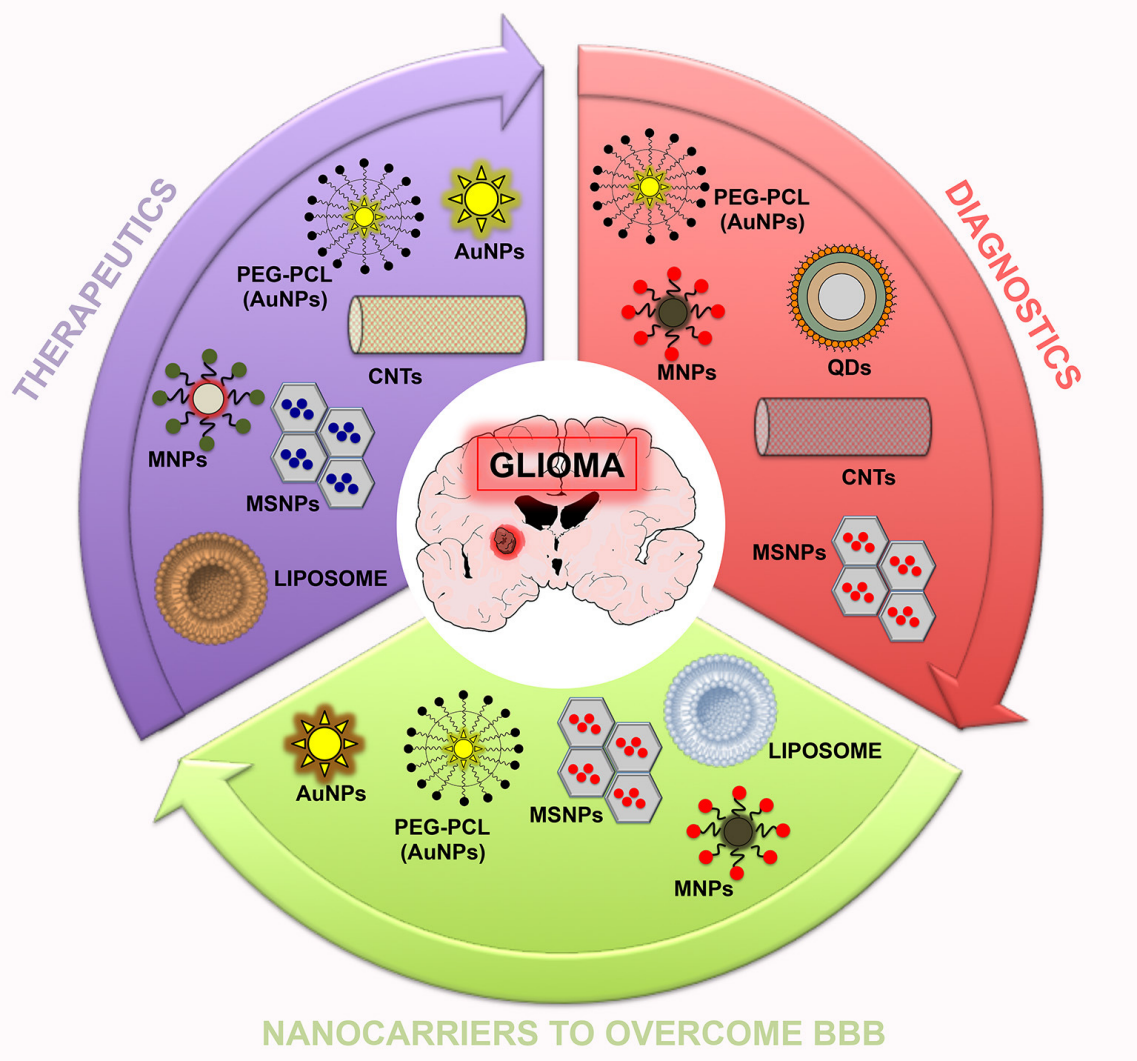
Summary scheme of nanoparticles potentially useful in theranostic nanomedicine for glioma. In this scheme, three sets (therapeutics, diagnostics, and nanocarriers to overcome the BBB) related to the potential application of nanoparticles are reported.

Conventional diagnostic and therapeutic agents showed improper bio-distribution and modest pharmacokinetics, leading to insufficient dissemination into tumors (Muthu et al., 2014a,b). In addition, they are non-specific and can accumulate in healthy organs, resulting in high toxicity. To overcome these issues, the nanotheranostic approach could be very useful. Different effective nanotheranostics brain cancer therapies have been recognized, but they need further investigation (Lakka and Rao, 2008; Xie et al., 2010; Keunen et al., 2011; Fan et al., 2014; Nance et al., 2014; Arranja et al., 2017). For instance, nanoparticle-enhanced imaging of the CNS at the subcellular level localizes more precisely the intracranial neoplasms area (Figure 2) (Bhojani et al., 2010). Also, nanoparticle-enhanced neuroimaging is very useful to understand physiological processes, including apoptosis, ischemia, inflammation, cell differentiation, and mitosis, representing the main tool for further research studies in neurodegenerative diseases and stroke (reviewed in Mattei and Rehman, 2015). To study physiological processes, many microscopic and macroscopic imaging modalities have been established. Microscopic methods require the invasive harvesting of tissues and imaging by cell-based assays (i.e., for apoptosis TUNEL, Annexin V, and Caspase Substrate Based Assays) (Cen et al., 2008). Macroscopic imaging modalities, by contrast, visualize apoptosis in living subjects in non-invasive modality. To date, to study these physiological processes, various *in vivo* molecular imaging technologies have been used, including Radiolabeled Small Molecular Probes, optical imaging probes, MRI agents, and multiple-modality methods. Microscopic and macroscopic imaging strategies improved the understanding of various physiological processes, or pathologies in preclinical and clinical studies (Zeng et al., 2015).

Unfortunately, the BBB still represents a limitation for nanotheranostic delivery (Wilhelm and Krizbai, 2014). To facilitate *in vitro* studies of drug delivery to the brain, promising *in vitro* BBB models have been developed based on primary or immortalized cells or on the culture of brain endothelial cells (Wilhelm and Krizbai, 2014; Helms et al., 2016). Valid models can be obtained using primary porcine brain endothelial cells and rodent co-culture models, which are characterized by low paracellular permeability and functional efflux transporters, mimicking the *in vivo* physiological complexity of the *in vivo* BBB. These include triple co-culture (brain endothelial cells with pericytes and astrocytes), dynamic, and microfluidic models, but these models are not suitable for rapid analyses. Great efforts have been made to deliver diagnostic agents and drugs into the brain. Thanks to recent advances in BBB research, new approaches have been exploited. Strategies able to pass through the BBB and reach the brain include viral vectors (characterized by high gene transfection efficiency), exosomes, brain permeability enhancers, and nanoparticles (Dong, 2018).

For example, Pilkington et al., in one of these *in vitro* BBB models, tested the properties of chitosyme nanoparticulate structures on BBB integrity, analyzing the tight junction proteins (ZO-1, occludin) and effects on the extra cellular matrix (Pilkington et al., 2014). In additional studies, paclitaxel (PTX) were constructed with a cyclic Arg-Gly-Asp (RGD) peptide as a targeting ligand to pass through the BBB by a targeting method. The nanocarriers were tested on a 3D glioma spheroid of glioma cells grown on agarose and showed targeted accumulation into tumor spheroids and excellent infiltration compared with conventional nanocarriers, suggesting a potential use in therapeutic approaches (Jiang et al., 2013). The theranostic nanosystems are combined with the targeting agent that identifies definite targets of brain cancer cells and binds to and internalizes via a specific mechanism. Several nanomaterials, including Gold Nanoparticles (AuNPs) and Quantum Dots (QDs) have intrinsic diagnostic/therapeutic properties (Muthu et al., 2014a; Sonali et al., 2018).

## Gold Nanoparticles

Gold Nanoparticles designed from gold cores represent a new system for theranostic systems (Table 2). They are biocompatible and are usually prepared as spheres, wire, rods, cubes, and cages. AuNPs, like other inorganic nanoparticles, trigger oxidative stress and subsequent cytotoxic effects. The spheroidal AuNPs ultraviolet (UV) absorption is at 520 nm, while the gold nanorods absorption is in the Infrared radiation (690–900 nm). These intrinsic optical characteristic allow AuNPs to be utilized as multifarious theranostic drugs for clinical applications (Xie et al., 2010; Kumar et al., 2013). AuNPs showed a diagnostic property, tunable core size, low toxicity, surface plasmon absorption and ease of fabrication, and light-scattering properties (Fan et al., 2014). AuNPs have been widely studied; for example, Melancon et al. formulated multi-utility gold-based nanoshells with optical and magnetic activities, which were additionally conjugated with targeting moiety and studied as an approach for head and neck cancer (Melancon et al., 2011). It has been shown that AuNPs improve the treatment of gliomas; for instance, the use of AuNPs-combined radiotherapy promoted long-term survival with respect to radiation therapy alone (Hainfeld et al., 2013; Joh et al., 2013). In another study, a theranostic system for cancer treatment, which was able to reduce the cytotoxic effect on normal cells, has been developed based on the use of gold nanoparticles surface-functionalized with a paclitaxel drug and biotin receptor. Two categories, AuNPs-4 and AuNPs-5, were investigated for their peculiar interaction with cancer cells. These nanoparticles were tested against the immortalized NIH3T3 cells, and it was suggested that the AuNPs-5 was more efficient (Heo et al., 2012). In addition, AuNPs represent an encouraging candidate for tumor margin detection, improving the surgery resection of brain cancers (Tzeng and Green, 2013). An *in-vitro* study on brain tumor cell lines showed a strong amelioration in uptake studies of targeted particles with respect to non-targeted formulations (Dixit et al., 2015b). Recently, matrix metalloproteinase-2-sensitive gold-gelatin nanoparticles were developed; RGD and octarginine were used as targeting ligands to pass through the BBB, allowing a pH-triggered release to the glioma-specific area. Indeed, it has been reported that gold nanotheranostic targeted specific tumor areas since it is able to co-localize within neovessels (Ruan et al., 2015). Gold theranostic micelles coated with polyethylene glycol-polycaprolactone (PEG-PCL) exhibited radiosensitizing efficacy for GBM therapy and can be used as a novel contrasting agent for both MRI and CT studies (Sun et al., 2016).

**Table 2:** Nanoparticles examples with some characteristics.

**Nanoparticles**	**Biosafety**	**Size**	**References**
Gold nanoparticles	Low cytotoxicity	2–60 nm	Fan et al., 2014
Magnetic nanoparticles	Potential cytotoxicity	7–20 nm	Alphandéry et al., 2015
Quantum dots	Potential cytotoxicity	2–50 nm	Onoshima et al., 2015
Carbon nanotubes	Potential cytotoxicity	0.4–40 nm	Wang et al., 2012
Mesoporous silica nanoparticles	Low cytotoxicity	20–25 nm	Wang et al., 2015

*Different nanoparticles and the respective biosafety and size*.

## Magnetic Nanoparticles

Recently, Magnetic Nanoparticles (MNPs) have been introduced as potential nanocarriers in targeted drug delivery at the tumor area, having the further benefit of MRI traceability (Table 2). The magnetic response (iron oxide core) ameliorates the magnetic targeted delivery (Pankhurst et al., 2003; Frimpong and Hilt, 2010). Interestingly, it has been shown that intravenous administration of these particles is able to reach the cancer site in an animal model. Recently, Chertok et al. reported that magnetic nanoparticles are a useful tool for magnetically enhanced accumulation in brain tumors and for non-invasive MRI screening. This accumulation can be sharply improved with magnetic targeting, as confirmed by MRI (Chertok et al., 2008). A recent *in-vivo* study suggested the potential clinical application of these nanotheranostics since MNPs overpass the BBB in a reversible way, and the substance can reach the targeted site (Lammers et al., 2015; Tabatabaei et al., 2015). Since 2013, NanoTherm® therapy has been established as a new procedure for the focal treatment of solid tumors (Rivera Gil et al., 2010). In this procedure, magnetic nanoparticles are introduced in the tumor or in the resection cavity wall. These particles are then heated by an alternating magnetic field, determining cancer cells death. Nanoparticles are particles of iron oxide, suspended in water, with a diameter of about 15 nm. After the *in vivo* engraftment, they agglomerate and remain the tissue to be treated. An alternating magnetic field then induces the particles to generate heat. Depending on the temperatures reached in the tumor site or in individual remaining cancer cells in the resection cavity wall, and the length of treatment, cancer cells are destroyed or made more sensitive to concomitant radiotherapy or chemotherapy^1^ (Alphandéry et al., 2015).

## Quantum Dots

Quantum Dots (QDs) are nanoscale (<10 nm) inorganic semiconductor nanocrystals, which represent a potential candidate for theranostic purposes (Table 2). They emit light which wavelength can be tuned on the basis of their shape, composition, and size. Cadmium selenide/Zinc sulfide-based QDs are the most used nanomaterials for diagnostic purposes. They have a CdSe core that is overcoated with layers of ZnS (Zhang et al., 2017). Furthermore, to gain affinity and target the cancer site, the surface of the QDs can be covalently or non-covalently conjugated with targeting probes, such as various antibodies, peptides, nucleic acids, folate aptamers, and other small molecules. One of the most suitable methods for conjugating targeting molecules on the surface of QDs is represented by the technique of avidin-biotin cross-linking (Tian et al., 2011; Onoshima et al., 2015). QDs can be conjugated with cancer cell-specific ligands, including HER2 (Ahmed et al., 2017), highly expressed in glioblastoma, CD44, proteins, antibodies, folic acid, and so on. Interestingly, QDs can be combined into paramagnetic liposomal designs containing RGD peptides and utilized as a diagnostic tool in tumor angiogenesis using MRI (Volkov, 2015). QDs in theranostic showed a clinical potential limit, due to their potential toxicity in humans (Derfus et al., 2004; Kirchner et al., 2005). To overcome this problem, further investigation is necessary to design biocompatible, excretable, surface-modified QDs (Onoshima et al., 2015).

## Carbon Nanotubes

Carbon Nanotubes (CNTs) are composed of different layers of graphene sheets, which form a cylindrical shape (Table 2). CNTs can be considered as allotropes of carbon with poor biocompatibility and slow biodegradation (Singh et al., 2016). CNTs are useful for theranostic applications since they can ameliorate the effect of chemotherapeutic agents and are translatable to clinical applications (Shapira et al., 2011; Singh et al., 2016). Once CNTs reach the targeted cells, they can interact with DNA and proteins, altering cellular signaling, or the mechanism of other therapeutic approaches (Ren et al., 2012; Chakrabarti et al., 2015). The intrinsic NIR light-absorption characteristic of CNTs has been exploited to eliminate tumor cells *in vitro*, whereas their NIR photoluminescence property has been utilized in cell imaging. In an interesting study, it has been reported that *i.v*. administration of single-walled carbon nanotubes (SWCNTs) as photo luminescent probes is a valid tool for *in vivo* tumor imaging, suggesting that SWCNTs could be used for theranostic applications. Moreover, CNTs are able to improve the chemotherapy effect in brain tumors (Robinson et al., 2010). In fact, recently, gold-coated surface-modified CNTs were established as optical nanotheranostic probes, which exhibited high potential as imaging tracers but had poor clinical potential due to slower biodegradation (and subsequent toxicity), as shown in *in vivo* nanotheranostic studies (Wang et al., 2012). However, CNTs may trigger adverse effects, such as lipid peroxidation, that induce inflammation and cell damage (Shapira et al., 2011; Singh et al., 2016).

## Mesoporous Silica Nanoparticles

Mesoporous Silica Nanoparticles (MSNPs) are also emerging drug delivery systems. MSNPs are thoroughly investigated and used in diagnostics because of their tunable shape and size and since their wide surface area facilitates a high drug loading (Table 2). Numerous drugs, including paclitaxel, camptothecin, methotrexate, colchicine, and cysteine, have been encapsulated in MSNPs. These encapsulated anticancer drugs are able to precisely cause the death of tumor cells (Gary-Bobo et al., 2012; Mamaeva et al., 2013). Thanks to the hexagonal structure, MSNPs can incorporate numerous functional components of an ideal theranostic approach in a single object, with different regions for the contrasting agent, therapeutic moiety, and biomolecular ligand. In addition, MSNPs are identified as safe materials by the FDA and are approved for evaluation in clinical studies. Scientists have utilized silica to integrate both IONPs and QDs, in order to create a hybrid with both optical and magnetic properties. MSNPs are biocompatible and biodegradable materials for nanotheranostic applications. MSNPs that dissolved silica can be absorbed by the biological system, metabolize, and be excreted through urine in the form of silicic acid or oligomeric silica species (Chen et al., 2013; Wang et al., 2015). Biomolecular targeting agents, proteins and peptides, are conjugated to the surface of MSNPs to ameliorate cancer treatment efficacy. Indeed, the surface of MSNPs was conjugated with a Tf peptide to enhance the detection of brain glioma cells (Cheng et al., 2010; Feng et al., 2016). Due to their efficient drug-loading capability, rugged nature, elevated biodegradation in the body, and diverse functionalization, MSNPs are widely used as tracers in MRIs or contrast agents in ultrasounds for accurate targeting, and they show positive results for brain cancer detection (Feng et al., 2016). In an interesting study, mesenchymal stem cells were engineered with MSNPs to treat and diagnose orthotopic glioblastomas. In particular, the intracerebral injection of engineered stem cells significantly improved the survival of rats with U87MG xenografts. This effect was concomitant with a reduction in tumor growth and proliferation and microvascular density. In GSC1 xenografts, intra-tumoral injections of Ad-hMSCs depleted the tumor cell population and induced migration of resident microglial cells (Pacioni et al., 2017). Nanotheranostics therapy was administered systemically to the mice and allowed *in vivo* imaging via MRI, NIR fluorescence, and PET; moreover, it exhibited high specificity for the glioma site (Huang et al., 2013).

## Nanoparticles for GB Treatment

Numerous nanostructured delivery systems have been established for brain tumor delivery, and, on the basis of their composition and nature, they can be divided into organic NPS and inorganic NPS (Kumar et al., 2016; Di Martino et al., 2017). Organic NPS (i.e., liposomes, polymeric nanoparticles, lipid nanoparticles, and micelles), compared with the “free” drugs, were able to efficiently cross the BBB, with favored distribution in the brain, in both *in vitro* and *in vivo* studies (Danhier et al., 2015; Chen et al., 2016; Kuo and Cheng, 2016; Liu et al., 2016; Qu et al., 2016; Wu et al., 2016; Belhadj et al., 2017; Chai et al., 2017; Graverini et al., 2018; Jhaveri et al., 2018) (Table 3). The main advantages of inorganic NPS (mesoporous silica nanoparticles, gold nanoparticles, iron oxide nanoparticles, and quantum dots) are their resistance to enzymatic degradation, robustness, and interesting intrinsic characteristics (Nam et al., 2013). For the treatment of GB, different kind of NPS (lipidic, magnetic, liposomal, fluorescent, and polymeric) have already been designed in order to cross the BBB, and these take into account active, passive, and stimuli-targeting perspectives (Cheng et al., 2014; Saraiva et al., 2016; Miranda et al., 2017; Aparicio-Blanco and Torres-Suárez, 2018). Theranostic nanoparticles represent a new technological concept that provides a combination of inorganic and organic nanoparticles to acquire synergistic characteristics in a single nanoparticle, exploiting the drug delivery by organic NPS and imaging by inorganic NPS. Theranostic nanoparticles can be used to limit toxicity due to a high and invasive dosage, improving patient outcomes. Recently, a combined chemo-photothermal targeted treatment of gliomas within one nanoparticle was developed. A targeting peptide was synthesized and characterized. In particular, as a therapeutic component, Doxorubicin was chosen, and, as a drug and diagnostic delivery system, a modified mesoporous silica-coated graphene nanosheet (GSPI) was chosen. The doxorubicin-loaded GSPI-based system showed heat-stimulating, pH-responsive, and sustained release properties. The *in vitro* results were encouraging; glioma cells showed a higher rate of death and strong GSPI accumulation (Lee et al., 2011). Targeting AuNPs with two or more receptor-binding peptides for glioblastoma treatment have been established (Dixit et al., 2015a). AuNPs conjugated with peptides (EGF and transferrin) and loaded with the photosensitizer phthalocyanine 4 (Pc 4) displayed synergistic effects in human glioma cells, concomitant with a high accumulation in the brain tumor area compared to AuNPs alone (Dixit et al., 2015a). Many *in vitro* studies reported positive effects; however, *in vivo* investigations based on theranostic NPS concepts are necessary to translate into clinical practice (Schmieder et al., 2008; Jokerst and Gambhir, 2011; Sailor and Park, 2012). *In vitro* and *in vitro* studies for GB treatment have been reported, and include gold nanoparticles, curcumin-loaded RDP-liposomes, curcumin-loaded PLGA-DSPE-PEG nanoparticles, chitosan-based nanoparticles, iron oxide nanoparticles coated with a chitosan-PEG-polyethyleneimine copolymer, hyaluronic acid conjugated liposomes, and others (Dixit et al., 2015a,b; Orunoglu et al., 2017; Zhao et al., 2018). Finally, magnetic nanoparticles and Nanotherm theranostic technology have been successfully applied in glioblastoma patients in 27 different European countries with double median survival in 59 patients (reviewed in Xie et al., 2018).

**Table 3:** Examples of clinical trials performed using nanoparticles drugs for gliomas.

**Drugs**	**Diseases**	**Phase**	**Clinical Trial**
ABI-009 (nab-rapamycin)	Recurrent high-grade glioma; Newly diagnosed glioblastoma	II	NCT03463265
NL CPT-11 (Nanoliposomal CPT-11)	Recurrent high-grade glioma	I completed	NCT00734682
Ferumoxytol	Recurrent high-grade glioma	I	NCT00769093
9-ING-41	Glioblastoma	II	NCT03678883
Pegylated Liposomal Doxorubicine + Temozolomide	Glioblastoma And diffuse intristic pontine glioma	II completed	*NCT00944801*
SGT-53	Recurrent glioblastoma	II	NCT02340156
Myocet	Refractory or relapsed malignant glioma in children/adolescent	I	NCT02861222

*Drugs, disease, and clinical trials with relative phase*.

All these studies reported high efficiency against glioblastoma also in *in vivo* investigations, thus indicating a promising application in diagnosis and concomitantly in therapeutic approaches, which results from significant accumulation in the brain tumor regions. NPS are poorly investigated in clinical trials (Andronescu and Grumezescu, 2017). The main limitation for using nanotechnology to diagnose and treat cancer is due to its inability to effectively contain and regulate the activity of NPS inside the body, comprising toxicity, biodistribution, and pharmacokinetics (Wicki et al., 2015; Bregoli et al., 2016; Hare et al., 2017).

## Conclusion and Perspectives

In the last years, the field of “theranostic medicine” has gained increasing interest in order to improve diagnostic and therapeutic interventions by nanotechnology resources that exploit a combined approach (Figure 1). A nanoparticle should contain a therapeutic agent combined with a tracer to help monitor the effect of the therapy as well. Theranostic is considered a potential candidate for targeted therapy and personalized medicine because you can follow the specific behavior of each tumor concomitant with a substantial increase in the efficacy of the anticancer drugs. This monitoring during the treatment course is a non-invasive method that, through the use of theranostic strategies, allows for the implementation of the individualization of therapeutic regimens based on each patient's response.

Overall, on light of the published investigations, nanotechnology research may be a potential and valid treatment of CNS pathologies, especially brain cancers (Figure 2), helping to address the main issues encountered: unclear tumoral margins, neurotoxicity of adjuvant therapies, fibrosis and immunological responses to intracranial devices, vascular anastomosis, multidrug resistance, BBB blockage, and tumor cell-specificity response to pharmacological treatments. Nanotheranostics, indeed, have shown to be a valid option for malignant brain cancers. Thus, in this review, we reported that nanotheranostics may represent a solid approach to be adopted in brain cancer management. The field of theranostics is pretty new, but considerable efforts have been made in order to develop theranostic nanoparticles for cancer therapy and targeted imaging. Advantages of theranostic nanoparticles include high biosafety, prolonged half-life into the circulatory system, concomitant loading of therapeutic and contrast agents, small size, high surface functionalization, and the ability to perform concomitantly diagnosis/monitoring and therapeutic approaches in real-time. Theranostic NPS allow a specific release of cargo in the affected site, targeting overexpressed proteins and receptors on brain cancer cells. These functions can facilitate the progress of innovative drugs in both preclinical and clinical phases.

Recently, multifunctional applications and combined approaches with personalized medicine applications have increased the hope in a successful clinical translation. Currently, as mentioned above, the only theranostic tool approved for use in the clinical treatment of GBM in Europe is NanoTherm® (Shi et al., 2017).

Overall, the goal is that multifunctional nanomedicine is an efficient, targeted *in vivo* drug delivery without systemic toxicity, and the therapeutic efficacy and the dosage can be precisely measured with low or absent invasivity.

However, in order to translate the experimental studies to clinical trials, further investigations are necessary, particularly to understand the low drug-loading capacity and to optimize the drug concentrations that reach the targeted area, and many factors need to be optimized simultaneously for the best clinical outcome.

*In vitro* and *in vivo* studies optimized to correctly evaluate toxicity, biodistribution, and pharmacokinetics of NPS are strongly requested to test the safety and efficacy of these nanomaterials in clinical studies. Moreover, the complexity of some nanoparticle designs and the high production costs contribute to the lower clinical uptake of NPS (Hare et al., 2017). The major efforts in the field of NPS should be directed toward bridging the gap between preclinical studies and the clinical phase.

The goal would be a better outcome for the patients thanks to the constant monitoring prior and during treatment, which allows for personalized cancer planning with predictable side effects. These multifunctional modern applications may increase a patient's life expectancy and life quality. It is highly probably that, in the near future, the field of nanotheranostics will emerge and become part of the conventional therapy and diagnostic approaches for brain cancer and other type of cancers.

## Author Contributions

All authors listed have made a substantial, direct and intellectual contribution to the work, and approved it for publication.

### Conflict of Interest

The authors declare that the research was conducted in the absence of any commercial or financial relationships that could be construed as a potential conflict of interest.

## Footnotes

^1^NanoTherm® therapy. Available online at: https://www.magforce.com/en/home/our_therapy/

## References

[B1] Agrawal P. Singh R. P. Sonali Kumari L. Sharma G. Koch B.. (2017a). TPGS-chitosan cross-linked targeted nanoparticles for effective brain cancer therapy. Mater. Sci. Eng. C Mater. Biol. Appl. 74, 167–176. 10.1016/j.msec.2017.02.008 28254282

[B2] Agrawal P. Sonali Singh R. P. Sharma G. Mehata A. K. Singh S.. (2017b). Bioadhesive micelles of d-α-tocopherol polyethylene glycol succinate 1000: synergism of chitosan and transferrin in targeted drug delivery. Colloids Surf. B Biointerfaces 152, 277–288. 10.1016/j.colsurfb.2017.01.021 28122295

[B3] Ahmed N. Brawley V. Hegde M. Bielamowicz K. Kalra M. Landi D.. (2017). HER2-specific chimeric antigen receptor-modified virus-specific t cells for progressive glioblastoma: a phase 1 dose-escalation trial. JAMA Oncol. 3, 1094–1101. 10.1001/jamaoncol.2017.0184 28426845 PMC5747970

[B4] Ahmed N. Fessi H. Elaissari A. (2012). Theranostic applications of nanoparticles in cancer. Drug Discov. Today 17, 928–934. 10.1016/j.drudis.2012.03.010 22484464

[B5] Ai H. (2011). Layer-by-layer capsules for magnetic resonance imaging and drug delivery. Adv. Drug Deliv. Rev. 63, 772–788. 10.1016/j.addr.2011.03.013 21554908

[B6] Al-Jamal W. T. Kostarelos K. (2011). Liposomes: from a clinically established drug delivery system to a nanoparticle platform for theranostic nanomedicine. Acc. Chem. Res. 44, 1094–1104. 10.1021/ar200105p 21812415

[B7] Alphandéry E. Grand-Dewyse P. Lefèvre R. Mandawala C. Durand-Dubief M. (2015). Cancer therapy using nanoformulated substances: scientific, regulatory and financial aspects. Expert Rev. Anticancer Ther. 15, 1233–1255. 10.1586/14737140.2015.1086647 26402250

[B8] Andronescu E. Grumezescu A. M. (2017). Nanostructures for Drug Delivery. Bucharest: Micro and Nano Technologies.

[B9] Ang C. Y. Tan S. Y. Lu Y. Bai L. Li M. Li P.. (2014). “Turn-on” fluorescence probe integrated polymer nanoparticles for sensing biological thiol molecules. Sci. Rep. 4:7057. 10.1038/srep07057 25394758 PMC4231329

[B10] Antonosante A. d'Angelo M. Castelli V. Catanesi M. Iannotta D. Giordano A.. (2018). The involvement of PPARs in the peculiar energetic metabolism of tumor cells. Int. J. Mol. Sci. 19:1907. 10.3390/ijms19071907 29966227 PMC6073339

[B11] Aparicio-Blanco J. Torres-Suárez A.-I. (2018). Towards tailored management of malignant brain tumors with nanotheranostics. Acta Biomater. 73, 52–63. 10.1016/j.actbio.2018.04.029 29678675

[B12] Arranja A. G. Pathak V. Lammers T. Shi Y. (2017). Tumor-targeted nanomedicines for cancer theranostics. Pharmacol. Res. 115, 87–95. 10.1016/j.phrs.2016.11.014 27865762 PMC5412956

[B13] Bahadur S. Sahu A. K. Baghel P. Saha S. (2019). Current promising treatment strategy for glioblastoma multiform: a review. Oncol. Rev. 13:417. 10.4081/oncol.2019.417 31410248 PMC6661528

[B14] Behin A. Hoang-Xuan K. Carpentier A. F. Delattre J.-Y. (2003). Primary brain tumours in adults. Lancet 361, 323–331. 10.1016/S0140-6736(03)12328-8 12559880

[B15] Belhadj Z. Zhan C. Ying M. Wei X. Xie C. Yan Z.. (2017). Multifunctional targeted liposomal drug delivery for efficient glioblastoma treatment. Oncotarget 8, 66889–66900. 10.18632/oncotarget.17976 28978003 PMC5620143

[B16] Benoy I. H. Salgado R. Van Dam P. Geboers K. Van Marck E. Scharpé S.. (2004). Increased serum interleukin-8 in patients with early and metastatic breast cancer correlates with early dissemination and survival. Clin. Cancer Res. 10, 7157–7162. 10.1158/1078-0432.CCR-04-0812 15534087

[B17] Bhojani M. S. Van Dort M. Rehemtulla A. Ross B. D. (2010). Targeted imaging and therapy of brain cancer using theranostic nanoparticles. Mol. Pharm. 7, 1921–1929. 10.1021/mp100298r 20964352 PMC3291122

[B18] Boas U. Heegaard P. M. H. (2004). Dendrimers in drug research. Chem. Soc. Rev. 33, 43–63. 10.1039/b309043b 14737508

[B19] Bregoli L. Movia D. Gavigan-Imedio J. D. Lysaght J. Reynolds J. Prina-Mello A. (2016). Nanomedicine applied to translational oncology: a future perspective on cancer treatment. Nanomedicine. 12, 81–103. 10.1016/j.nano.2015.08.006 26370707

[B20] Cai W. Rao J. Gambhir S. S. Chen X. (2006). How molecular imaging is speeding up antiangiogenic drug development. Mol. Cancer Ther. 5, 2624–2633. 10.1158/1535-7163.MCT-06-0395 17121909

[B21] Cantley L. C. Neel B. G. (1999). New insights into tumor suppression: PTEN suppresses tumor formation by restraining the phosphoinositide 3-kinase/AKT pathway. Proc. Natl. Acad. Sci. U.S.A. 96, 4240–4245. 10.1073/pnas.96.8.4240 10200246 PMC33561

[B22] Caracciolo V. Laurenti G. Romano G. Carnevale V. Cimini A. M. Crozier-Fitzgerald C.. (2012). Flavopiridol induces phosphorylation of AKT in a human glioblastoma cell line, in contrast to siRNA-mediated silencing of Cdk9: implications for drug design and development. Cell Cycle 11, 1202–1216. 10.4161/cc.11.6.19663 22391209

[B23] Cen H. Mao F. Aronchik I. Fuentes R. J. Firestone G. L. (2008). DEVD-NucView488: a novel class of enzyme substrates for real-time detection of caspase-3 activity in live cells. FASEB J. 22, 2243–2252. 10.1096/fj.07-099234 18263700

[B24] Chai Z. Hu X. Wei X. Zhan C. Lu L. Jiang K.. (2017). A facile approach to functionalizing cell membrane-coated nanoparticles with neurotoxin-derived peptide for brain-targeted drug delivery. J. Control. Release 264, 102–111. 10.1016/j.jconrel.2017.08.027 28842313

[B25] Chakrabarti M. Kiseleva R. Vertegel A. Ray S. K. (2015). Carbon nanomaterials for drug delivery and cancer therapy. J. Nanosci. Nanotechnol. 15, 5501–5511. 10.1166/jnn.2015.10614 26369109

[B26] Chakravarti A. Loeffler J. S. Dyson N. J. (2002). Insulin-like growth factor receptor I mediates resistance to anti-epidermal growth factor receptor therapy in primary human glioblastoma cells through continued activation of phosphoinositide 3-kinase signaling. Cancer Res. 62, 200–207. 11782378

[B27] Chang Y. Meng X. Zhao Y. Li K. Zhao B. Zhu M.. (2011). Novel water-soluble and pH-responsive anticancer drug nanocarriers: doxorubicin-PAMAM dendrimer conjugates attached to superparamagnetic iron oxide nanoparticles (IONPs). J. Colloid Interface Sci. 363, 403–409. 10.1016/j.jcis.2011.06.086 21821262

[B28] Chen N.-T. Cheng S.-H. Souris J. S. Chen C.-T. Mou C.-Y. Lo L.-W. (2013). Theranostic applications of mesoporous silica nanoparticles and their organic/inorganic hybrids. J. Mater. Chem. B 1:3128 10.1039/c3tb20249f 32260912

[B29] Chen Z. Lai X. Song S. Zhu X. Zhu J. (2016). Nanostructured lipid carriers based temozolomide and gene co-encapsulated nanomedicine for gliomatosis cerebri combination therapy. Drug Deliv. 23, 1369–1373. 10.3109/10717544.2015.1038857 26017099

[B30] Cheng S.-H. Lee C.-H. Chen M.-C. Souris J. S. Tseng F.-G. Yang C.-S. (2010). Tri-functionalization of mesoporous silica nanoparticles for comprehensive cancer theranostics—the trio of imaging, targeting and therapy. J. Mater. Chem. 20:6149 10.1039/c0jm00645a

[B31] Cheng Y. Morshed R. A. Auffinger B. Tobias A. L. Lesniak M. S. (2014). Multifunctional nanoparticles for brain tumor imaging and therapy. Adv. Drug Deliv. Rev. 66, 42–57. 10.1016/j.addr.2013.09.006 24060923 PMC3948347

[B32] Chertok B. Moffat B. A. David A. E. Yu F. Bergemann C. Ross B. D.. (2008). Iron oxide nanoparticles as a drug delivery vehicle for MRI monitored magnetic targeting of brain tumors. Biomaterials 29, 487–496. 10.1016/j.biomaterials.2007.08.050 17964647 PMC2761681

[B33] Choi K. Y. Liu G. Lee S. Chen X. (2012). Theranostic nanoplatforms for simultaneous cancer imaging and therapy: current approaches and future perspectives. Nanoscale 4, 330–342. 10.1039/C1NR11277E 22134683 PMC3629960

[B34] Cimini A. d'Angelo M. Benedetti E. D'Angelo B. Laurenti G. Antonosante A.. (2017). Flavopiridol: an old drug with new perspectives? Implication for development of new drugs. J. Cell. Physiol. 232, 312–322. 10.1002/jcp.25421 27171480

[B35] Cole A. J. Yang V. C. David A. E. (2011). Cancer theranostics: the rise of targeted magnetic nanoparticles. Trends Biotechnol. 29, 323–332. 10.1016/j.tibtech.2011.03.001 21489647 PMC3210200

[B36] Danhier F. Messaoudi K. Lemaire L. Benoit J.-P. Lagarce F. (2015). Combined anti-Galectin-1 and anti-EGFR siRNA-loaded chitosan-lipid nanocapsules decrease temozolomide resistance in glioblastoma: *in vivo* evaluation. Int. J. Pharm. 481, 154–161. 10.1016/j.ijpharm.2015.01.051 25644286

[B37] Derfus A. M. Chan W. C. W. Bhatia S. N. (2004). Probing the cytotoxicity of semiconductor quantum dots. Nano Lett. 4, 11–18. 10.1021/nl0347334 28890669 PMC5588688

[B38] Di Martino A. Guselnikova O. A. Trusova M. E. Postnikov P. S. Sedlarik V. (2017). Organic-inorganic hybrid nanoparticles controlled delivery system for anticancer drugs. Int. J. Pharm. 526, 380–390. 10.1016/j.ijpharm.2017.04.061 28465052

[B39] Dixit S. Miller K. Zhu Y. McKinnon E. Novak T. Kenney M. E.. (2015a). Dual receptor-targeted theranostic nanoparticles for localized delivery and activation of photodynamic therapy drug in glioblastomas. Mol. Pharm. 12, 3250–3260. 10.1021/acs.molpharmaceut.5b00216 26198693 PMC4564323

[B40] Dixit S. Novak T. Miller K. Zhu Y. Kenney M. E. Broome A.-M. (2015b). Transferrin receptor-targeted theranostic gold nanoparticles for photosensitizer delivery in brain tumors. Nanoscale 7, 1782–1790. 10.1039/C4NR04853A 25519743 PMC4437576

[B41] Dong X. (2018). Current strategies for brain drug delivery. Theranostics 8, 1481–1493. 10.7150/thno.21254 29556336 PMC5858162

[B42] Duncan R. (2003). The dawning era of polymer therapeutics. Nat. Rev. Drug Discov. 2, 347–360. 10.1038/nrd1088 12750738

[B43] Fan Z. Fu P. P. Yu H. Ray P. C. (2014). Theranostic nanomedicine for cancer detection and treatment. J. Food Drug Anal. 22, 3–17. 10.1016/j.jfda.2014.01.001 24673900 PMC9359153

[B44] Fattahi H. Laurent S. Liu F. Arsalani N. Vander Elst L. Muller R. N. (2011). Magnetoliposomes as multimodal contrast agents for molecular imaging and cancer nanotheragnostics. Nanomedicine. 6, 529–544. 10.2217/nnm.11.14 21542690

[B45] Feng Y. Panwar N. Tng D. J. H. Tjin S. C. Wang K. Yong K.-T. (2016). The application of mesoporous silica nanoparticle family in cancer theranostics. Coord. Chem. Rev. 319, 86–109. 10.1016/j.ccr.2016.04.019

[B46] Fidoamore A. Cristiano L. Antonosante A. d'Angelo M. Di Giacomo E. Astarita C.. (2016). Glioblastoma stem cells microenvironment: the paracrine roles of the niche in drug and radioresistance. Stem Cells Int. 2016:6809105. 10.1155/2016/6809105 26880981 PMC4736577

[B47] Frezza C. Gottlieb E. (2009). Mitochondria in cancer: not just innocent bystanders. Semin. Cancer Biol. 19, 4–11. 10.1016/j.semcancer.2008.11.008 19101633

[B48] Frimpong R. A. Hilt J. Z. (2010). Magnetic nanoparticles in biomedicine: synthesis, functionalization and applications. Nanomedicine 5, 1401–1414. 10.2217/nnm.10.114 21128722

[B49] Gary-Bobo M. Hocine O. Brevet D. Maynadier M. Raehm L. Richeter S.. (2012). Cancer therapy improvement with mesoporous silica nanoparticles combining targeting, drug delivery and PDT. Int. J. Pharm. 423, 509–515. 10.1016/j.ijpharm.2011.11.045 22178618

[B50] Gatenby R. A. Gillies R. J. (2004). Why do cancers have high aerobic glycolysis? Nat. Rev. Cancer 4, 891–899. 10.1038/nrc1478 15516961

[B51] Gaya A. Rees J. Greenstein A. Stebbing J. (2002). The use of temozolomide in recurrent malignant gliomas. Cancer Treat. Rev. 28, 115–120. 10.1053/ctrv.2002.0261 12297119

[B52] Golombek S. K. May J.-N. Theek B. Appold L. Drude N. Kiessling F.. (2018). Tumor targeting via EPR: strategies to enhance patient responses. Adv. Drug Deliv. Rev. 130, 17–38. 10.1016/j.addr.2018.07.007 30009886 PMC6130746

[B53] Graverini G. Piazzini V. Landucci E. Pantano D. Nardiello P. Casamenti F.. (2018). Solid lipid nanoparticles for delivery of andrographolide across the blood-brain barrier: *in vitro* and *in vivo* evaluation. Colloids Surf. B Biointerfaces 161, 302–313. 10.1016/j.colsurfb.2017.10.062 29096375

[B54] Gürsel D. B. Shin B. J. Burkhardt J.-K. Kesavabhotla K. Schlaff C. D. Boockvar J. A. (2011). Glioblastoma stem-like cells-biology and therapeutic implications. Cancers. 3, 2655–2666. 10.3390/cancers3022655 21796273 PMC3142771

[B55] Hainfeld J. F. Smilowitz H. M. O'Connor M. J. Dilmanian F. A. Slatkin D. N. (2013). Gold nanoparticle imaging and radiotherapy of brain tumors in mice. Nanomedicine. 8, 1601–1609. 10.2217/nnm.12.165 23265347 PMC3657324

[B56] Hare J. I. Lammers T. Ashford M. B. Puri S. Storm G. Barry S. T. (2017). Challenges and strategies in anti-cancer nanomedicine development: an industry perspective. Adv. Drug Deliv. Rev. 108, 25–38. 10.1016/j.addr.2016.04.025 27137110

[B57] Harrington K. J. Mohammadtaghi S. Uster P. S. Glass D. Peters A. M. Vile R. G.. (2001). Effective targeting of solid tumors in patients with locally advanced cancers by radiolabeled pegylated liposomes. Clin. Cancer Res. 7, 243–254. 11234875

[B58] Helms H. C. Abbott N. J. Burek M. Cecchelli R. Couraud P.-O. Deli M. A.. (2016). *In vitro* models of the blood–brain barrier: an overview of commonly used brain endothelial cell culture models and guidelines for their use. J Cerebral Blood Flow Metab. 36, 862–890. 10.1177/0271678X16630991 26868179 PMC4853841

[B59] Heo D. N. Yang D. H. Moon H.-J. Lee J. B. Bae M. S. Lee S. C.. (2012). Gold nanoparticles surface-functionalized with paclitaxel drug and biotin receptor as theranostic agents for cancer therapy. Biomaterials 33, 856–866. 10.1016/j.biomaterials.2011.09.064 22036101

[B60] Hsu S. P. C. Kuo J. S. Chiang H.-C. Wang H.-E. Wang Y.-S. Huang C.-C.. (2018). Temozolomide, sirolimus and chloroquine is a new therapeutic combination that synergizes to disrupt lysosomal function and cholesterol homeostasis in GBM cells. Oncotarget 9, 6883–6896. 10.18632/oncotarget.23855 29467937 PMC5805523

[B61] Huang X. Zhang F. Wang H. Niu G. Choi K. Y. Swierczewska M.. (2013). Mesenchymal stem cell-based cell engineering with multifunctional mesoporous silica nanoparticles for tumor delivery. Biomaterials 34, 1772–1780. 10.1016/j.biomaterials.2012.11.032 23228423 PMC3538138

[B62] Jhaveri A. Deshpande P. Pattni B. Torchilin V. (2018). Transferrin-targeted, resveratrol-loaded liposomes for the treatment of glioblastoma. J. Control. Release 277, 89–101. 10.1016/j.jconrel.2018.03.006 29522834 PMC5911193

[B63] Jiang X. Sha X. Xin H. Xu X. Gu J. Xia W.. (2013). Integrin-facilitated transcytosis for enhanced penetration of advanced gliomas by poly(trimethylene carbonate)-based nanoparticles encapsulating paclitaxel. Biomaterials 34, 2969–2979. 10.1016/j.biomaterials.2012.12.049 23380351

[B64] Joh D. Y. Sun L. Stangl M. Al Zaki A. Murty S. Santoiemma P. P.. (2013). Selective targeting of brain tumors with gold nanoparticle-induced radiosensitization. PLoS ONE 8:e62425. 10.1371/journal.pone.0062425 23638079 PMC3640092

[B65] Jokerst J. V. Gambhir S. S. (2011). Molecular imaging with theranostic nanoparticles. Acc. Chem. Res. 44, 1050–1060. 10.1021/ar200106e 21919457 PMC3196845

[B66] Kelkar S. S. Reineke T. M. (2011). Theranostics: combining imaging and therapy. Bioconjug. Chem. 22, 1879–1903. 10.1021/bc200151q 21830812

[B67] Kenny G. D. Kamaly N. Kalber T. L. Brody L. P. Sahuri M. Shamsaei E.. (2011). Novel multifunctional nanoparticle mediates siRNA tumour delivery, visualisation and therapeutic tumour reduction *in vivo*. J. Control. Release 149, 111–116. 10.1016/j.jconrel.2010.09.020 20888381

[B68] Keunen O. Johansson M. Oudin A. Sanzey M. Rahim S. A. A. Fack F.. (2011). Anti-VEGF treatment reduces blood supply and increases tumor cell invasion in glioblastoma. Proc. Natl. Acad. Sci. U.S.A. 108, 3749–3754. 10.1073/pnas.1014480108 21321221 PMC3048093

[B69] Kievit F. M. Zhang M. (2011). Cancer nanotheranostics: improving imaging and therapy by targeted delivery across biological barriers. Adv. Mater. Weinheim 23, H217–247. 10.1002/adma.201102313 21842473 PMC3397249

[B70] Kirchner C. Liedl T. Kudera S. Pellegrino T. Muñoz Javier A. Gaub H. E.. (2005). Cytotoxicity of colloidal CdSe and CdSe/ZnS nanoparticles. Nano Lett. 5, 331–338. 10.1021/nl047996m 15794621

[B71] Kondo T. Setoguchi T. Taga T. (2004). Persistence of a small subpopulation of cancer stem-like cells in the C6 glioma cell line. Proc. Natl. Acad. Sci. U.S.A. 101, 781–786. 10.1073/pnas.0307618100 14711994 PMC321758

[B72] Koo Y.-E. L. Reddy G. R. Bhojani M. Schneider R. Philbert M. A. Rehemtulla A.. (2006). Brain cancer diagnosis and therapy with nanoplatforms. Adv. Drug Deliv. Rev. 58, 1556–1577. 10.1016/j.addr.2006.09.012 17107738

[B73] Kroemer G. Pouyssegur J. (2008). Tumor cell metabolism: cancer's achilles' heel. Cancer Cell 13, 472–482. 10.1016/j.ccr.2008.05.005 18538731

[B74] Kroonen J. Nguyen-Khac M. T. Deprez M. Rogister B. Robe P. (2008). [Glioblastoma, an example of translational research?]. Rev. Med. Liege 63, 251–256. 18669189

[B75] Kumar A. Lee J.-Y. Kim H.-S. (2016). Selective fluorescence sensing of 3,5-dinitrosalicylic acid based on pyrenesulfonamide-functionalized inorganic/organic hybrid nanoparticles. J. Industr. Eng. Chem. 44, 82–89. 10.1016/j.jiec.2016.08.010

[B76] Kumar A. Zhang X. Liang X.-J. (2013). Gold nanoparticles: emerging paradigm for targeted drug delivery system. Biotechnol. Adv. 31, 593–606. 10.1016/j.biotechadv.2012.10.002 23111203

[B77] Kuo Y.-C. Cheng S.-J. (2016). Brain targeted delivery of carmustine using solid lipid nanoparticles modified with tamoxifen and lactoferrin for antitumor proliferation. Int. J. Pharm. 499, 10–19. 10.1016/j.ijpharm.2015.12.054 26721730

[B78] Lakka S. S. Rao J. S. (2008). Antiangiogenic therapy in brain tumors. Expert Rev. Neurother. 8, 1457–1473. 10.1586/14737175.8.10.1457 18928341 PMC2656359

[B79] Lammers T. Kiessling F. Hennink W. E. Storm G. (2010). Nanotheranostics and image-guided drug delivery: current concepts and future directions. Mol. Pharm. 7, 1899–1912. 10.1021/mp100228v 20822168

[B80] Lammers T. Koczera P. Fokong S. Gremse F. Ehling J. Vogt M.. (2015). Theranostic USPIO-loaded microbubbles for mediating and monitoring blood-brain barrier permeation. Adv. Funct. Mater. 25, 36–43. 10.1002/adfm.201401199 25729344 PMC4340520

[B81] Lee J. E. Lee N. Kim T. Kim J. Hyeon T. (2011). Multifunctional mesoporous silica nanocomposite nanoparticles for theranostic applications. Acc. Chem. Res. 44, 893–902. 10.1021/ar2000259 21848274

[B82] Lindner L. H. Hossann M. (2010). Factors affecting drug release from liposomes. Curr. Opin. Drug Discov. Devel. 13, 111–123. 20047152

[B83] Liong M. Lu J. Kovochich M. Xia T. Ruehm S. G. Nel A. E.. (2008). Multifunctional inorganic nanoparticles for imaging, targeting, and drug delivery. ACS Nano 2, 889–896. 10.1021/nn800072t 19206485 PMC2751731

[B84] Liu G. Wang Z. Lu J. Xia C. Gao F. Gong Q.. (2011). Low molecular weight alkyl-polycation wrapped magnetite nanoparticle clusters as MRI probes for stem cell labeling and *in vivo* imaging. Biomaterials 32, 528–537. 10.1016/j.biomaterials.2010.08.099 20869767

[B85] Liu X. Madhankumar A. B. Miller P. A. Duck K. A. Hafenstein S. Rizk E.. (2016). MRI contrast agent for targeting glioma: interleukin-13 labeled liposome encapsulating gadolinium-DTPA. Neuro-oncology 18, 691–699. 10.1093/neuonc/nov263 26519740 PMC4827043

[B86] Lo S.-T. Kumar A. Hsieh J.-T. Sun X. (2013). Dendrimer nanoscaffolds for potential theranostics of prostate cancer with a focus on radiochemistry. Mol. Pharm. 10, 793–812. 10.1021/mp3005325 23294202 PMC3599782

[B87] Lu J. Ma S. Sun J. Xia C. Liu C. Wang Z.. (2009). Manganese ferrite nanoparticle micellar nanocomposites as MRI contrast agent for liver imaging. Biomaterials 30, 2919–2928. 10.1016/j.biomaterials.2009.02.001 19230966

[B88] Mamaeva V. Sahlgren C. Lindén M. (2013). Mesoporous silica nanoparticles in medicine–recent advances. Adv. Drug Deliv. Rev. 65, 689–702. 10.1016/j.addr.2012.07.018 22921598

[B89] Mattei T. A. Rehman A. A. (2015). “Extremely minimally invasive”: recent advances in nanotechnology research and future applications in neurosurgery. Neurosurg. Rev. 38, 27–37; discussion 37. 10.1007/s10143-014-0566-2 25173621

[B90] Melancon M. P. Lu W. Zhong M. Zhou M. Liang G. Elliott A. M.. (2011). Targeted multifunctional gold-based nanoshells for magnetic resonance-guided laser ablation of head and neck cancer. Biomaterials 32, 7600–7608. 10.1016/j.biomaterials.2011.06.039 21745689 PMC3195359

[B91] Mendes M. Sousa J. J. Pais A. Vitorino C. (2018). Targeted theranostic nanoparticles for brain tumor treatment. Pharmaceutics 10:E181. 10.3390/pharmaceutics10040181 30304861 PMC6321593

[B92] Merkel O. M. Mintzer M. A. Librizzi D. Samsonova O. Dicke T. Sproat B. (2010). Triazine dendrimers as non-viral vectors for *in vitro* and *in vivo* RNAi: the effects of peripheral groups and core structure on biological activity. Mol. Pharm. 7, 969–983. 10.1021/mp100101s 20524664 PMC2914146

[B93] Mintzer M. A. Grinstaff M. W. (2011). Biomedical applications of dendrimers: a tutorial. Chem. Soc. Rev. 40, 173–190. 10.1039/B901839P 20877875

[B94] Miranda A. Blanco-Prieto M. J. Sousa J. Pais A. Vitorino C. (2017). Breaching barriers in glioblastoma. Part II: Targeted drug delivery and lipid nanoparticles. Int. J. Pharm. 531, 389–410. 10.1016/j.ijpharm.2017.07.049 28801108

[B95] Muthu M. S. Leong D. T. Mei L. Feng S.-S. (2014a). Nanotheranostics - application and further development of nanomedicine strategies for advanced theranostics. Theranostics 4, 660–677. 10.7150/thno.8698 24723986 PMC3982135

[B96] Muthu M. S. Mei L. Feng S.-S. (2014b). Nanotheranostics: advanced nanomedicine for the integration of diagnosis and therapy. Nanomedicine 9, 1277–1280. 10.2217/nnm.14.83 25204816

[B97] Na K. Lee S. A. Jung S. H. Shin B. C. (2011). Gadolinium-based cancer therapeutic liposomes for chemotherapeutics and diagnostics. Colloids Surfaces B Biointerfaces 84, 82–87. 10.1016/j.colsurfb.2010.12.019 21251801

[B98] Nam J. Won N. Bang J. Jin H. Park J. Jung S.. (2013). Surface engineering of inorganic nanoparticles for imaging and therapy. Adv. Drug Deliv. Rev. 65, 622–648. 10.1016/j.addr.2012.08.015 22975010

[B99] Nam L. Coll C. Erthal L. de la Torre C. Serrano D. Martínez-Máñez R.. (2018). Drug delivery nanosystems for the localized treatment of glioblastoma multiforme. Materials. 11:779. 10.3390/ma11050779 29751640 PMC5978156

[B100] Nance E. Zhang C. Shih T.-Y. Xu Q. Schuster B. S. Hanes J. (2014). Brain-penetrating nanoparticles improve paclitaxel efficacy in malignant glioma following local administration. ACS Nano 8, 10655–10664. 10.1021/nn504210g. 25259648 PMC4212792

[B101] Newton H. B. (2010). Overview of the molecular genetics and molecular chemotherapy of GBM, in Glioblastoma, ed Ray S. K. (New York, NY: Springer New York), 1–42. 10.1007/978-1-4419-0410-2_1

[B102] Nicholas M. K. Lukas R. V. Jafri N. F. Faoro L. Salgia R. (2006). Epidermal growth factor receptor - mediated signal transduction in the development and therapy of gliomas. Clin. Cancer Res. 12, 7261–7270. 10.1158/1078-0432.CCR-06-0874 17189397

[B103] Onoshima D. Yukawa H. Baba Y. (2015). Multifunctional quantum dots-based cancer diagnostics and stem cell therapeutics for regenerative medicine. Adv. Drug Deliv. Rev. 95, 2–14. 10.1016/j.addr.2015.08.004 26344675

[B104] Orunoglu M. Kaffashi A. Pehlivan S. B. Sahin S. Söylemezoglu F. Oguz K. K.. (2017). Effects of curcumin-loaded PLGA nanoparticles on the RG2 rat glioma model. Mater. Sci. Eng. C 78, 32–38. 10.1016/j.msec.2017.03.292 28575990

[B105] Pacioni S. D'Alessandris Q. G. Giannetti S. Morgante L. Coccè V. Bonomi A.. (2017). Human mesenchymal stromal cells inhibit tumor growth in orthotopic glioblastoma xenografts. Stem Cell Res. Ther. 8:53. 10.1186/s13287-017-0516-3 28279193 PMC5345323

[B106] Pan B. Cui D. Sheng Y. Ozkan C. Gao F. He R.. (2007). Dendrimer-modified magnetic nanoparticles enhance efficiency of gene delivery system. Cancer Res. 67, 8156–8163. 10.1158/0008-5472.CAN-06-4762 17804728

[B107] Pankhurst Q. A. Connolly J. Jones S. K. Dobson J. (2003). Applications of magnetic nanoparticles in biomedicine. J. Phys. D Appl. Phys. 36, R167–R181. 10.1088/0022-3727/36/13/201

[B108] Park J.-H. Ryu C. H. Kim M. J. Jeun S.-S. (2015). Combination therapy for gliomas using temozolomide and interferon-beta secreting human bone marrow derived mesenchymal stem cells. J. Korean Neurosurg. Soc. 57, 323–328. 10.3340/jkns.2015.57.5.323 26113958 PMC4479712

[B109] Peer D. Karp J. M. Hong S. Farokhzad O. C. Margalit R. Langer R. (2007). Nanocarriers as an emerging platform for cancer therapy. Nat. Nanotechnol. 2, 751–760. 10.1038/nnano.2007.387 18654426

[B110] Petersen A. L. Binderup T. Jølck R. I. Rasmussen P. Henriksen J. R. Pfeifer A. K.. (2012). Positron emission tomography evaluation of somatostatin receptor targeted 64Cu-TATE-liposomes in a human neuroendocrine carcinoma mouse model. J. Control. Release 160, 254–263. 10.1016/j.jconrel.2011.12.038 22245688

[B111] Pilkington G. J. Maherally Z. Jassam S. Barbu E. Fillmore H. (2014). An all human 3D *in vitro* model of the blood brain barrier in nanoparticle delivery and cancer metastasis studies. Neuro-Oncology 16:iii33 10.1093/neuonc/nou208.39

[B112] Piquer J. Llácer J. L. Rovira V. Riesgo P. Rodriguez R. Cremades A. (2014). Fluorescence-guided surgery and biopsy in gliomas with an exoscope system. Biomed Res. Int. 2014:207974. 10.1155/2014/207974 24971317 PMC4055357

[B113] Puputti M. Tynninen O. Sihto H. Blom T. Mäenp,ää H. Isola J.. (2006). Amplification of KIT, PDGFRA, VEGFR2, and EGFR in gliomas. Mol. Cancer Res. 4, 927–934. 10.1158/1541-7786.MCR-06-0085 17189383

[B114] Pysz M. A. Gambhir S. S. Willmann J. K. (2010). Molecular imaging: current status and emerging strategies. Clin. Radiol. 65, 500–516. 10.1016/j.crad.2010.03.011 20541650 PMC3150531

[B115] Qu J. Zhang L. Chen Z. Mao G. Gao Z. Lai X.. (2016). Nanostructured lipid carriers, solid lipid nanoparticles, and polymeric nanoparticles: which kind of drug delivery system is better for glioblastoma chemotherapy? Drug Deliv. 23, 3408–3416. 10.1080/10717544.2016.1189465 27181462

[B116] Ren J. Shen S. Wang D. Xi Z. Guo L. Pang Z.. (2012). The targeted delivery of anticancer drugs to brain glioma by PEGylated oxidized multi-walled carbon nanotubes modified with angiopep-2. Biomaterials 33, 3324–3333. 10.1016/j.biomaterials.2012.01.025 22281423

[B117] Rivera Gil P. Hühn D. del Mercato L. L. Sasse D. Parak W. J. (2010). Nanopharmacy: inorganic nanoscale devices as vectors and active compounds. Pharmacol. Res. 62, 115–125. 10.1016/j.phrs.2010.01.009 20097288

[B118] Robinson J. T. Welsher K. Tabakman S. M. Sherlock S. P. Wang H. Luong R.. (2010). High performance *in vivo* near-IR (>1 μm) imaging and photothermal cancer therapy with carbon nanotubes. Nano Res. 3, 779–793. 10.1007/s12274-010-0045-1 21804931 PMC3143483

[B119] Rock K. McArdle O. Forde P. Dunne M. Fitzpatrick D. O'Neill B.. (2012). A clinical review of treatment outcomes in glioblastoma multiforme–the validation in a non-trial population of the results of a randomised Phase III clinical trial: has a more radical approach improved survival? Br. J. Radiol. 85, e729–733. 10.1259/bjr/83796755 22215883 PMC3487092

[B120] Ruan S. He Q. Gao H. (2015). Matrix metalloproteinase triggered size-shrinkable gelatin-gold fabricated nanoparticles for tumor microenvironment sensitive penetration and diagnosis of glioma. Nanoscale 7, 9487–9496. 10.1039/c5nr01408e 25909483

[B121] Sailor M. J. Park J.-H. (2012). Hybrid nanoparticles for detection and treatment of cancer. Adv. Mater. 24, 3779–3802. 10.1002/adma.201200653 22610698 PMC3517011

[B122] Saraiva C. Praça C. Ferreira R. Santos T. Ferreira L. Bernardino L. (2016). Nanoparticle-mediated brain drug delivery: overcoming blood-brain barrier to treat neurodegenerative diseases. J. Control. Release 235, 34–47. 10.1016/j.jconrel.2016.05.044 27208862

[B123] Schlessinger J. (2000). Cell signaling by receptor tyrosine kinases. Cell 103, 211–225. 10.1016/S0092-8674(00)00114-8 11057895

[B124] Schmieder A. H. Caruthers S. D. Zhang H. Williams T. A. Robertson J. D. Wickline S. A.. (2008). Three-dimensional MR mapping of angiogenesis with alpha5beta1(alpha nu beta3)-targeted theranostic nanoparticles in the MDA-MB-435 xenograft mouse model. FASEB J. 22, 4179–4189. 10.1096/fj.08-112060 18697838 PMC2614609

[B125] Shapira A. Livney Y. D. Broxterman H. J. Assaraf Y. G. (2011). Nanomedicine for targeted cancer therapy: towards the overcoming of drug resistance. Drug Resist. Updat. 14, 150–163. 10.1016/j.drup.2011.01.003 21330184

[B126] Shi J. Kantoff P. W. Wooster R. Farokhzad O. C. (2017). Cancer nanomedicine: progress, challenges and opportunities. Nat. Rev. Cancer 17, 20–37. 10.1038/nrc.2016.108 27834398 PMC5575742

[B127] Silantyev F. Falzone L. Libra M. Gurina O. I. Kardashova K. S. Nikolouzakis T. K.. (2019). Current and future trends on diagnosis and prognosis of glioblastoma: from molecular biology to proteomics. Cells 8:863. 10.3390/cells8080863 31405017 PMC6721640

[B128] Singh R. P. Sharma G. Sonali Singh S. Kumar M. Pandey B. L.. (2016). Vitamin E TPGS conjugated carbon nanotubes improved efficacy of docetaxel with safety for lung cancer treatment. Colloids Surf. B Biointerfaces 141, 429–442. 10.1016/j.colsurfb.2016.02.011 26895505

[B129] Slettenaar V. I. F. Wilson J. L. (2006). The chemokine network: a target in cancer biology? Adv. Drug Deliv. Rev. 58, 962–974. 10.1016/j.addr.2006.03.012 16996642

[B130] Sonali Agrawal P. Singh R. P. Rajesh C. V. Singh S. Vijayakumar M. R. (2016a). Transferrin receptor-targeted vitamin E TPGS micelles for brain cancer therapy: preparation, characterization and brain distribution in rats. Drug Deliv. 23, 1788–1798. 10.3109/10717544.2015.1094681 26431064

[B131] Sonali Singh R. P. Sharma G. Kumari L. Koch B. Singh S. (2016b). RGD-TPGS decorated theranostic liposomes for brain targeted delivery. Colloids Surf. B Biointerfaces 147, 129–141. 10.1016/j.colsurfb.2016.07.058 27497076

[B132] Sonali Singh R. P. Singh N. Sharma G. Vijayakumar M. R. Koch B. (2016c). Transferrin liposomes of docetaxel for brain-targeted cancer applications: formulation and brain theranostics. Drug Deliv. 23, 1261–1271. 10.3109/10717544.2016.1162878 26961144

[B133] Sonali Viswanadh M. K. Singh R. P. Agrawal P. Mehata A. K. Pawde D. M.. (2018). Nanotheranostics: emerging strategies for early diagnosis and therapy of brain cancer. Nanotheranostics 2, 70–86. 10.7150/ntno.21638 29291164 PMC5743839

[B134] Stambolic V. Suzuki A. de la Pompa J. L. Brothers G. M. Mirtsos C. Sasaki T.. (1998). Negative regulation of PKB/Akt-dependent cell survival by the tumor suppressor PTEN. Cell 95, 29–39. 10.1016/S0092-8674(00)81780-8 9778245

[B135] Su H. Liu Y. Wang D. Wu C. Xia C. Gong Q.. (2013). Amphiphilic starlike dextran wrapped superparamagnetic iron oxide nanoparticle clsuters as effective magnetic resonance imaging probes. Biomaterials 34, 1193–1203. 10.1016/j.biomaterials.2012.10.056 23168385

[B136] Sun D. (2010). Nanotheranostics: integration of imaging and targeted drug delivery. Mol. Pharm. 7:1879. 10.1021/mp1003652 21128687

[B137] Sun L. Joh D. Y. Al-Zaki A. Stangl M. Murty S. Davis J. J.. (2016). Theranostic application of mixed gold and superparamagnetic iron oxide nanoparticle micelles in glioblastoma multiforme. J. Biomed. Nanotechnol. 12, 347–356. 10.1166/jbn.2016.2173 27305768 PMC4942305

[B138] Tabatabaei S. N. Girouard H. Carret A.-S. Martel S. (2015). Remote control of the permeability of the blood-brain barrier by magnetic heating of nanoparticles: a proof of concept for brain drug delivery. J. Control. Release 206, 49–57. 10.1016/j.jconrel.2015.02.027 25724273

[B139] Tanaka N. Kanatani S. Tomer R. Sahlgren C. Kronqvist P. Kaczynska D.. (2017). Whole-tissue biopsy phenotyping of three-dimensional tumours reveals patterns of cancer heterogeneity. Nat. Biomed. Eng. 1, 796–806. 10.1038/s41551-017-0139-0 31015588

[B140] Tandel G. S. Biswas M. Kakde O. G. Tiwari A. Suri H. S. Turk M. (2019). A review on a deep learning perspective in brain cancer classification. Cancers. 11:E111 10.3390/cancers11010111 30669406 PMC6356431

[B141] Tennant D. A. Frezza C. MacKenzie E. D. Nguyen Q. D. Zheng L. Selak M. A.. (2009). Reactivating HIF prolyl hydroxylases under hypoxia results in metabolic catastrophe and cell death. Oncogene 28, 4009–4021. 10.1038/onc.2009.250 19718054

[B142] Thakkar J. P. Dolecek T. A. Horbinski C. Ostrom Q. T. Lightner D. D. Barnholtz-Sloan J. S.. (2014). Epidemiologic and molecular prognostic review of glioblastoma. Cancer Epidemiol. Biomarkers Prev. 23, 1985–1996. 10.1158/1055-9965.EPI-14-0275 25053711 PMC4185005

[B143] Tian B. Al-Jamal W. T. Al-Jamal K. T. Kostarelos K. (2011). Doxorubicin-loaded lipid-quantum dot hybrids: surface topography and release properties. Int. J. Pharm. 416, 443–447. 10.1016/j.ijpharm.2011.01.057 21315141

[B144] Tong R. Cheng J. (2007). Anticancer polymeric nanomedicines. Poly. Rev. 47, 345–381. 10.1080/15583720701455079

[B145] Tzeng S. Y. Green J. J. (2013). Therapeutic nanomedicine for brain cancer. Ther. Deliv. 4, 687–704. 10.4155/tde.13.38 23738667 PMC3838881

[B146] Uchida N. Buck D. W. He D. Reitsma M. J. Masek M. Phan T. V.. (2000). Direct isolation of human central nervous system stem cells. Proc. Natl. Acad. Sci. U.S.A. 97, 14720–14725. 10.1073/pnas.97.26.14720 11121071 PMC18985

[B147] Vilos C. Velasquez L. A. (2012). Therapeutic strategies based on polymeric microparticles. J. Biomed. Biotechnol. 2012:672760. 10.1155/2012/672760 22665988 PMC3363323

[B148] Volkov Y. (2015). Quantum dots in nanomedicine: recent trends, advances and unresolved issues. Biochem. Biophys. Res. Commun. 468, 419–427. 10.1016/j.bbrc.2015.07.039 26168726

[B149] Wang D. Lin B. Ai H. (2014). Theranostic nanoparticles for cancer and cardiovascular applications. Pharm. Res. 31, 1390–1406. 10.1007/s11095-013-1277-z 24595494

[B150] Wang H. Liu X. Wang Y. Chen Y. Jin Q. Ji J. (2015). Doxorubicin conjugated phospholipid prodrugs as smart nanomedicine platforms for cancer therapy. J. Mater. Chem. B 3, 3297–3305. 10.1039/C4TB01984A 32262324

[B151] Wang X. Wang C. Cheng L. Lee S.-T. Liu Z. (2012). Noble metal coated single-walled carbon nanotubes for applications in surface enhanced Raman scattering imaging and photothermal therapy. J. Am. Chem. Soc. 134, 7414–7422. 10.1021/ja300140c 22486413

[B152] Wang Y. Shim M. S. Levinson N. S. Sung H.-W. Xia Y. (2014). Stimuli-responsive materials for controlled release of theranostic agents. Adv. Funct. Mater. 24, 4206–4220. 10.1002/adfm.201400279 25477774 PMC4249693

[B153] Wei X. Chen X. Ying M. Lu W. (2014). Brain tumor-targeted drug delivery strategies. Acta Pharm. Sin. B 4, 193–201. 10.1016/j.apsb.2014.03.001 26579383 PMC4629063

[B154] Wesseling P. Capper D. (2018). WHO 2016 classification of gliomas. Neuropathol. Appl. Neurobiol. 44, 139–150. 10.1111/nan.12432 28815663

[B155] Wicki A. Witzigmann D. Balasubramanian V. Huwyler J. (2015). Nanomedicine in cancer therapy: challenges, opportunities, and clinical applications. J. Controlled Release 200, 138–157. 10.1016/j.jconrel.2014.12.030 25545217

[B156] Wilhelm I. Krizbai I. A. (2014). *In vitro* models of the blood-brain barrier for the study of drug delivery to the brain. Mol. Pharm. 11, 1949–1963. 10.1021/mp500046f 24641309

[B157] Wong A. J. Ruppert J. M. Bigner S. H. Grzeschik C. H. Humphrey P. A. Bigner D. S.. (1992). Structural alterations of the epidermal growth factor receptor gene in human gliomas. Proc. Natl. Acad. Sci. U.S.A. 89, 2965–2969. 10.1073/pnas.89.7.2965 1557402 PMC48784

[B158] Wu M. Fan Y. Lv S. Xiao B. Ye M. Zhu X. (2016). Vincristine and temozolomide combined chemotherapy for the treatment of glioma: a comparison of solid lipid nanoparticles and nanostructured lipid carriers for dual drugs delivery. Drug Deliv. 23, 2720–2725. 10.3109/10717544.2015.1058434 26203691

[B159] Xiao Q. Yang S. Ding G. Luo M. (2018). Anti-vascular endothelial growth factor in glioblastoma: a systematic review and meta-analysis. Neurol. Sci. 39, 2021–2031. 10.1007/s10072-018-3568-y 30327956

[B160] Xie J. Lee S. Chen X. (2010). Nanoparticle-based theranostic agents. Adv. Drug Deliv. Rev. 62, 1064–1079. 10.1016/j.addr.2010.07.009 20691229 PMC2988080

[B161] Xie J. Liu G. Eden H. S. Ai H. Chen X. (2011). Surface-engineered magnetic nanoparticle platforms for cancer imaging and therapy. Acc. Chem. Res. 44, 883–892. 10.1021/ar200044b 21548618 PMC3166427

[B162] Xie W. Guo Z. Gao F. Gao Q. Wang D. Liaw B.. (2018). Shape-, size- and structure-controlled synthesis and biocompatibility of iron oxide nanoparticles for magnetic theranostics. Theranostics 8, 3284–3307. 10.7150/thno.25220 29930730 PMC6010979

[B163] Yamanaka R. (2008). Cell- and peptide-based immunotherapeutic approaches for glioma. Trends Mol. Med. 14, 228–235. 10.1016/j.molmed.2008.03.003 18403264

[B164] Zeng W. Wang X. Xu P. Liu G. Eden H. S. Chen X. (2015). Molecular imaging of apoptosis: from micro to macro. Theranostics 5, 559–582. 10.7150/thno.11548 25825597 PMC4377726

[B165] Zhang B. Yang C. Gao Y. Wang Y. Bu C. Hu S.. (2017). Engineering quantum dots with different emission wavelengths and specific fluorescence lifetimes for spectrally and temporally multiplexed imaging of cells. Nanotheranostics 1, 131–140. 10.7150/ntno.18989 29071182 PMC5646722

[B166] Zhao M. Zhao M. Fu C. Yu Y. Fu A. (2018). Targeted therapy of intracranial glioma model mice with curcumin nanoliposomes. Int. J. Nanomedicine 13, 1601–1610. 10.2147/IJN.S157019 29588587 PMC5858816

[B167] Zong T. Mei L. Gao H. Cai W. Zhu P. Shi K.. (2014). Synergistic dual-ligand doxorubicin liposomes improve targeting and therapeutic efficacy of brain glioma in animals. Mol. Pharm. 11, 2346–2357. 10.1021/mp500057n 24893333

[B168] Zottel A. Videtič Paska A. Jovčevska I. (2019). Nanotechnology meets oncology: nanomaterials in brain cancer research, diagnosis and therapy. Materials. 12:1588. 10.3390/ma12101588 31096609 PMC6567262

