# Corrigendum: Theranostic Nanomedicine for Malignant Gliomas

**DOI:** 10.3389/fbioe.2019.00468

**Published:** 2020-01-30

**Authors:** Michele d'Angelo, Vanessa Castelli, Elisabetta Benedetti, Andrea Antonosante, Mariano Catanesi, Reyes Dominguez-Benot, Giuseppina Pitari, Rodolfo Ippoliti, Annamaria Cimini

**Affiliations:** ^1^Department of Life, Health and Environmental Sciences, University of L'Aquila, L'Aquila, Italy; ^2^Department of Biology, Sbarro Institute for Cancer Research and Molecular Medicine, Temple University, Philadelphia, PA, United States

**Keywords:** theranostic nanoplatform, brain tumors, targeted therapy, drug delivery, diagnosis

In the original article, we neglected to include the funder. This project has received funding from the **European Union's Horizon 2020 research and innovation programme** under the Marie Skłodowska-Curie **grant agreement No 713714** to **Reyes Dominguez-Benot**.

The authors apologize for this error and state that this does not change the scientific conclusions of the article in any way. The original article has been updated.

